# Decoys and Regulatory “Receptors” of the IL-1/Toll-Like Receptor Superfamily

**DOI:** 10.3389/fimmu.2013.00180

**Published:** 2013-07-09

**Authors:** Cecilia Garlanda, Federica Riva, Eduardo Bonavita, Stefania Gentile, Alberto Mantovani

**Affiliations:** ^1^Department of Inflammation and Immunology, Humanitas Clinical and Research Center, Rozzano, Italy; ^2^Department of Veterinary Science and Public Health, University of Milan, Milan, Italy; ^3^Department of Biotechnology and Translational Medicine, University of Milan, Rozzano, Milan, Italy

**Keywords:** cytokine, interleukin-1, inflammation, decoy receptor

## Abstract

Members of the IL-1 family play a key role in innate and adaptive immunity and in the pathogenesis of diverse diseases. Members of IL-1R like receptor (ILR) family include signaling molecules and negative regulators. The latter include decoy receptors (IL-1RII; IL-18BP) and “receptors” with regulatory function (TIR8/SIGIRR; IL-1RAcPb; DIGIRR). Structural considerations suggest that also TIGIRR-1 and IL-1RAPL may have regulatory function. The presence of multiple pathways of negative regulation of members of the IL-1/IL-1R family emphasizes the need for a tight control of members of this fundamental system.

## Introduction

IL-1R like receptors (ILRs) belong, together with Toll-like receptors (TLRs), to a superfamily of phylogenetically conserved proteins involved in innate immunity and inflammation (1–5). The common characteristic of the members of this family is the presence of a conserved domain in the cytoplasmic region, called TIR domain, originally defined as the Toll/IL-1-resistance and now generally assumed as an acronym for Toll/IL-1R domain. The TIR domain is involved in the activation of an evolutionarily conserved signaling pathway leading to NF-kB translocation to the nucleus and activation of protein kinases such as p38, c-Jun N-terminal kinases (JNKs), extracellular signal-regulated kinases (ERKs), and mitogen-activated protein kinases (mAPKs) (6). The ILR subfamily includes the receptors and the accessory proteins (AcP) for IL-1α (IL-1F1) and IL-1β (IL-1F2), IL-18/IL-1F4, IL-33/IL-F11, and other IL-1 family members (IL-36α/IL-1F6, IL-36β/IL-1F8, and IL-36γ/IL-1F9), which are involved in the initiation of an amplification cascade of innate resistance and inflammation and contribute to the activation and orientation of adaptive immunity (7–9). Some members of the family remain orphan receptors with still unknown ligands and functions. For instance, in the IL-1R subfamily, TIR8/SIGIRR, TIGIRR-1, and IL-1RAPL have no characterized ligands so far (2, 10, 11).

The activation of the ILR-dependent signaling cascade is tightly regulated. Indeed, the deregulated activation of these receptors, which lead to the production of proteins related to inflammation and immunity, potentially mediates damaging local and systemic inflammatory reactions. Several pathological conditions depend, at least in part, on the inflammatory potential of the IL-1 family members mentioned above. For instance, the IL-1 system represents a relevant therapeutic target in arthritis, type 2 diabetes, psoriasis, sepsis, ischemia and reperfusion, atherosclerosis, graft rejection, cancer (12–15). The regulatory mechanisms identified so far in the IL-1 system (ligands, receptors, signaling pathway) act extracellularly or intracellularly (16, 17). IL-1R antagonists (IL-1Ra)/IL-1F3 and IL-36Ra/IL-1F5 are polypeptide antagonists competing with IL-1 and IL-36α/IL-1F6, IL-36β/IL-1F8, and IL-36γ/IL-1F9, respectively, for receptor binding (3, 7, 18–20). IL-1RII lacks a signaling domain and by binding IL-1 prevents its interaction with a signaling receptor complex and therefore acts as a decoy, dominant-negative molecule, and scavenger. The negative regulator of ILR and TLR signaling, TIR8 (also known as SIGIRR), acts intracellularly. IRAK-M and MyD88s are intracellular negative regulators of ILRs and TLRs signaling (21, 22). Finally, ILR or TLR signaling proteins or transcription factors are targets of miRNAs, such as miR-155, miR-21, miR-146a, miR-132, miR-9, and miR-147, whose transcription is induced by inflammatory mediators [lipopolysaccharide (LPS), TNFα, IL-1 β] through NF-kB (23–25).

Here, we summarize our current understanding of the structure and function of negative regulatory receptors of the ILR family, in particular IL-1RII, which has served to defining the decoy receptor paradigm, and TIR8/SIGIRR, focusing on their regulatory roles in different pathological disorders dependent on ILRs and TLRs activity, and finally describe other largely uncharacterized members of the family with a negative regulatory potential, TIGIRR-1, IL-1RAPL, IL-1RAPb.

## The Decoy Receptor IL-1RII

### Gene and protein

**Figure 1:**
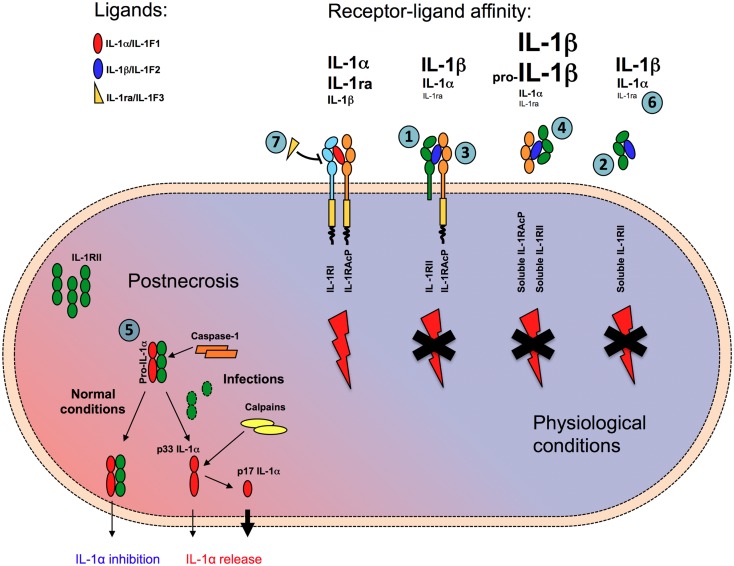
**Mechanisms of negative regulation mediated by IL-1RII**. IL-1RII negatively regulates IL-1 responses in multiple ways. In membrane-bound (1) or soluble form (2) IL-1RII acts as a decoy, capturing with high-affinity IL-1β and IL-1α, but not IL-1ra, and preventing their interaction with IL-1RI. IL-1RII acts as a dominant-negative influencing IL-1RI-IL-1RAcp signaling receptor complex formation (3). The interaction of ligand-bound soluble IL-1RII with soluble IL-1RAcP increases the affinity for both IL-1α and IL-1β by about two orders of magnitude (4). Finally, in cytosol soluble form, IL-1RII interacts with pro-IL-1α preventing its pro-inflammatory activity during necrosis, until caspase-1 cleaves IL-1RII and allows the secretion of IL-1α (5). Because of low affinity for IL-1RII, IL-1ra does not compete with IL-1α and IL-1β for the interaction with the decoy receptor (6), whereas it competes with IL-1α and IL-1β antagonizing their interaction with the signaling receptor IL-1RI (7).

The first IL-1R was cloned from murine and human T cells, whereas IL-1RII was identified soon after in B lymphocytes and myelomonocytic cells (26, 27). On the basis of their structures, IL-1RI and IL-1RII belong to the Ig-like superfamily of receptors, with the extracellular portion containing 3 Ig-like domains. The signaling IL-1R complex includes the type I IL-1 receptor (IL-1RI) and IL-1R AcP, which both have a cytoplasmic TIR domain (Figure 1).

The gene encoding IL-1RII is located on chromosome 2 (q12–22) in humans and in the centromeric region of chromosome 1 in mice (28), in cluster with IL-1RI and other members of the family (IL-33R, IL-18R, IL-36R). The type II receptor is highly conserved in evolution and is found in bony fish, where it functions to inhibit IL-1-induced inflammation (29). The third Ig domain of IL-1RII is homologous to the Ig domain of IL-18BP (30) and indeed, it has been suggested that IL-1RII and IL-18BP have a common ancestral gene and diverged at the level of fish (31). The IL-1RII locus spans about 38 kb of genomic DNA, of which about 21 kb contains the coding region. The exon structures of the extracellular portion of IL-1RII and IL-1RI receptors are identical and amino acid sequences share 28% homology. A single exon encodes the transmembrane region and a short cytoplasmic tail (29 amino acids) of IL-1RII, which has no TIR domain and does not signal (Figure 1). The human transcript encodes for a 386 amino acid glycosylated protein of 68 kDa, in contrast with IL-1RI which is a 80–85 kDa glycosylated protein and has a 213 amino acid cytoplasmic tail containing a TIR domain responsible for signaling (32). IL-1RII can be proteolytically processed and released in a soluble form, via the actions of a metalloproteinase, A Disintegrin and Metalloprotease 17 (ADAM17, also know as TACE) (33, 34). In addition, IL-1RII can be processed in a manner similar to Amyloid β protein precursor (APP), by alpha-, beta-, and gamma-secretase: the ectodomain is shed in an alpha-secretase-like manner, whereas the IL-1RII C-terminal fragment undergoes further intramembrane proteolysis by gamma-secretase (35). Finally, the aminopeptidase regulator of TNFR1 shedding (ARTS-1) has been implicated in IL-1RII shedding in basal condition and upon cell stimulation with phorbol myristate acetate (PMA) (36).

Wang et al. (37) solved the structure of IL-1 and IL-1RAcP in complex with the extracellular domain of IL-1RII and showed that the mode of interaction among IL-1β, IL-1RII, and IL-1RAcP and the overall structure are extremely similar to those of the signaling ligand-receptor complex (IL-1, IL-1R1, IL-1AcP).

### Mechanisms of negative regulation

Several lines of evidence are consistent with the view that the IL-1RII is a bonafide IL-1 decoy.

A first level of control is represented by the differential affinity of the signaling and decoy receptors for agonist or antagonist ligands of the IL-1 family (Figure 1). IL-1RI binds IL-1α with higher affinity than IL-1β (*K*_d_ ≈ 10^−10^ and 10^−9^ M, respectively) and IL-1ra with an affinity similar to that for IL-1α. By contrast, IL-1RII binds IL-1β and IL-1α with high affinity (*K*_d_ ≈ 10^−9^–10^−10^ and 10^−8^ M, respectively), but it binds IL-1ra at least 100 times less efficiently (38). Plasmon resonance analysis revealed that IL-1β has a slow off-rate from IL-1RII, whereas IL-1ra rapidly dissociates from IL-1RII but not from IL-1RI (39), in agreement with the need that the two regulators of IL-1 do not bind each other self defeating and frustrating their regulatory activity. By binding agonist ligands with high affinity without inducing signaling, IL-1RII acts as a molecular trap for IL-1 inhibiting its activity (27, 40) (Figure 1).

Second, IL-1RII also forms a complex with IL-1 and the IL-1RAcP. It therefore exerts a dominant-negative effect on the formation of a signaling receptor complex, by sequestering AcP, which is essential for signal transduction (41, 42) (Figure 1).

In addition, IL-1RII is also found in a soluble form, released from cells via the actions of a metalloproteinase (see above). Soluble IL-1RII is found in normal blood relatively at high concentrations, in the order of nanogram per milliliter. Cell-surface shedding is the major mechanism responsible of soluble IL-1RII generation, but in addition an alternatively spliced transcript encoding a soluble version of IL-1RII has been described (43). Soluble, but not cell-associated, IL-1RII binds pro-IL-1β and blocks its processing by IL-1-converting enzyme (ICE)/caspase-1 (38) (Figure 1). Soluble AcP, encoded by an alternatively spliced mRNA (44) and found at high levels in the circulation (300 ng/ml in humans), can interact with ligand-bound soluble IL-1RII, enhancing the latter’s affinity for IL-1α and IL-1β by two orders of magnitude, while not affecting the very low affinity for IL-1Ra (40, 45) (Figure 1). In mouse and monkey, the interaction between AcP and IL-1RII is required for high-affinity binding of IL-1β and effective inhibition (45). Thus, the interaction with AcP renders IL-1RII a much more effective inhibitor of IL-1.

Finally, a further mechanism of negative control of IL-1α by IL-1RII during necrosis has recently been proposed (46). The soluble form of IL-1RII has been detected in the cytosol in large amounts, possibly because the IL-1RII signal peptide is short and relatively weak. In line with previous reports on systemic sclerosis fibroblasts (47), in this cytosolic form, soluble IL-1RII interacts with pro-IL-1α (Figure 1). This interaction protects pro-IL-1α from cleavage by different enzymes (calpain, granzyme B, chymase, and elastase) normally involved in the generation of the active form (48, 49) and prevents IL-1α activity (46). This blockade would be abrogated by active caspase-1 (for instance during infections), which specifically cleaves IL-1RII, causing dissociation from IL-1α, calpain processing, and complete restoration of IL-1α activity after necrosis or during regulated secretion (Figure 1). Since IL-1RII is expressed by a limited set of cell types, in contrast with IL-1RI, which is widely expressed, this mechanism of negative regulation would be cell type specific. Thus, the activity of IL-1α during necrosis and sterile inflammation would be somehow restricted to cell types which do not express IL-1RII. For instance, the high inflammatory profile of vascular smooth muscle cells to necrosis, which is IL-1α-dependent (50), would be in agreement with low levels of IL-1RII. These findings would explain the tissue specificity of inflammatory damage during necrosis.

The anti-inflammatory role of IL-1RII has been demonstrated in different pathological conditions in animal models. Gene-targeted mice overexpressing IL-1RII under the control of the human keratin gene promoter were resistant to PMA-induced chronic skin inflammation (51). Recombinant IL-1RII delivered via implanted human keratinocytes overexpressing soluble IL-1RII played a protective role in a mouse model of collagen-induced arthritis (52) and intravenous administration of soluble IL-1RII significantly reduced joint swelling and erosion in a model of arthritis in rabbit (53). Gene transfer of a soluble IL-1RII-Ig fusion protein reduced allograft rejection and prolonged graft survival in a rat model of heart transplantation, reduced infiltrating macrophages, and CD4+ T cells, and lowered levels of TNF-α and TGF-β (54). Similarly, IL-1RII ameliorated experimental autoimmune myocarditis by blocking IL-1 and inhibiting production of the cytokines [IL-6, transforming growth factor-β, retinoic acid-related orphan nuclear receptor (RORγt) and IL-17] involved in the polarization of Th17 cells (55). Finally, in a mouse model of endometriosis, consisting of human endometrial tissue implanted in nude mice, human soluble IL-1-RII administered intraperitoneally reduced the growth and dissemination of endometrial implants and the expression of IL-1β-dependent inflammatory, angiogenic, and cell growth mediators (56).

In support of the view that IL-1RII is a professional anti-IL-1 molecule, Pox viruses have acquired and retained a soluble version of type II IL-1R, that plays a key role in the regulation of pathogenicity (57).

Thus, IL-1RII negatively regulates IL-1 responses in multiple complementary ways. In membrane-bound or soluble form IL-1RII acts as a decoy, capturing with high affinity IL-1, and preventing it from interacting with IL-1RI. It acts as a dominant-negative influencing IL-1RI-IL-1RAcp signaling receptor complex formation. The interaction of ligand-bound soluble IL-1RII with soluble IL-1RAcP increases the affinity for both IL-1α and IL-1β by about two orders of magnitude and makes IL-1RII a powerful inhibitor for both agonists. Finally, in cytosol soluble form, IL-1RII interacts with pro-IL-1α preventing its pro-inflammatory activity during necrosis.

### Expression

In contrast with IL-1RI, which is expressed by a large variety of cell types, IL-1RII is expressed by a limited set of cell types, which also often express IL-1RI: among leukocytes, IL-1RII is the predominant IL-1-binding protein found in monocytes, neutrophils, and B cells (26, 27, 40, 58). Monocyte differentiation to macrophages, in particular M2 or M2-like macrophages, is associated to increased expression of IL-1RII (58, 59). IL-1RII is also expressed by microglial cells, in particular upon stimulation with LPS and has been shown to regulate IL-1β actions by binding excess levels of this cytokine during brain inflammation (60). In addition, noradrenaline has been reported to upregulate IL-1RII in mixed microglia via β-adrenoceptor activation and downstream activation of protein kinase A and ERK, thus preventing IL-1β-induced neurotoxicity (61). Other stimuli involved in IL-1RII upregulation in the CNS include cerebral ischemia, kainic acid administration, and central administration of IL-1β (62).

T regulatory cells (Tregs) have been shown to express surface and soluble functional IL-1RII, as well as IL-1Ra mRNA. This property has been exploited for the purification of activated human FOXP3+ regulatory T cells from expansion cultures (63). Activated human Tregs rapidly up-regulated IL-1RII and were able to neutralize IL-1β, which suggests a physiological significance for the expression of IL-1 decoy receptor on Tregs (64).

Differential levels of IL-1RII have been described in osteoclasts. In particular, lower expression of IL-1RII has been detected in large osteoclasts compared to small osteoclasts, and this is in line with increased resorptive activity of large osteoclasts in response to IL-1 (65). IL-1RII is also expressed by basal epithelial cells of the skin (66), epithelium of endometrium (67), vagina and urethra, and chondrocytes. Endothelial cells and fibroblasts generally express only IL-1RI and AcP.

Surface and soluble IL-1RII expression is strongly enhanced by anti-inflammatory signals. Glucocorticoid hormones (GCs), prostaglandins, the anti-inflammatory T helper 2 (Th2) cytokines (IL-4 and IL-13), and IL-27 induced augmented surface expression and release of IL-1RII *in vitro*, in particular in myelomonocytic cells, and *in vivo* (27, 58, 68– 72). In particular, IL-4 and dexamethasone, by inducing IL-1RII, antagonized the prosurvival effect of IL-1 in neutrophils *in vitro* (27). IL-10 increased circulating soluble IL-1RII levels *in vivo* in mice. Aspirin increased IL-1RII release from mononuclear cell cultures *in vitro* and *in vivo* (73). IL-27 inhibited IL-1β-induced signaling in human macrophages by downregulating the expression of the signaling receptor IL-1RI, inducing expression of the receptor antagonist IL-1Ra, and by upregulating the expression of the decoy receptor IL-1RII (72). These data suggest that induction of IL-1RII contributes to the anti-inflammatory effect of these mediators.

In contrast, pro-inflammatory molecules inhibit IL-1RII expression. For instance, bacterial LPS caused a rapid shedding of surface IL-1RII in monocytes, followed by down-regulation of expression, whereas it stimulated the expression of IL-1RI, AcP and the adapter protein MyD88 (74). Interferon γ (IFN-γ) inhibited IL-1RII expression and release in myelomonocytic cells and counteracted IL-4-dependent upregulation of IL-1RII (71). In addition to LPS, chemoattractants such as formyl Meth-Leu-Phe (fMLP), reactive oxygen intermediates (ROI), TNF, and PMA caused rapid shedding of IL-1RII (33, 75, 76). PMA also induced alternatively spliced soluble IL-1RAcP (44). Thus, shedding of IL-1RII by circulating phagocytes and generation of alternatively spliced soluble IL-1RAcP induced by chemoattractants in the early steps of recruitment, could prepare cells to respond to IL-1 once they enter tissues.

Acetylated low density (ac-LDL) and very low density (VLDL) lipoprotein intracellular accumulation caused decreased IL-1RII mRNA and protein expression in macrophages *in vitro*. In agreement with these *in vitro* data, patients with familial combined hyperlipidemia showed decreased expression of IL-1RII in monocytes. Finally, IL-1RII expression in human atherosclerotic vessels was defective compared to non-atherosclerotic arteries (77).

Naturally circulating levels of soluble IL-1R type II are in the range of 5–10 ng/ml, although these can rise in certain chronic (78) or acute (79) inflammatory settings (see below), in part reflecting the activation of negative circuits of regulation of the cytokine action.

### IL-1RII in human pathological conditions: Diagnostic and therapeutic implications

High levels of soluble IL-1RII are normally present in plasma of healthy individuals. Defective or increased expression of tissue or body fluid levels of soluble IL-1RII have been described in diverse pathological conditions, ranging from critical conditions to autoimmune diseases, neuroinflammatory diseases and tumors.

Increased blood levels of soluble IL-1RII have been detected in critically ill patients with infectious conditions such as sepsis, acute meningococcal infection, experimental endotoxemia, operative trauma, or necrotizing enterocolitis in preterm infants (73, 80, 81). In critically ill patients, IL-1RII levels were elevated especially in severe, systemic infection and culture-positive infections. In patients with a marked systemic inflammatory response syndrome, further pronounced increase of circulating IL-1RII levels was observed in patients developing sepsis (80). Treatment with glucocorticoids further increased IL-1RII levels, suggesting that it potentially behaves as a biomarker for the activation of anti-inflammatory pathways or for responsiveness to anti-inflammatory agents. In acute meningococcal infections, increased soluble IL-1RII levels correlated with disease severity, in particular with endotoxemia, complement-activation, and shock (82). Increased IL-1RII levels were also observed in patients upon treatment with aspirin (73).

IL-1Ra and/or IL-1RII increased levels were also detected in sera of multiple sclerosis patients after steroid treatment for relapse (83) and in the cerebrospinal fluid of patients with Alzheimer’s disease, where it may be a marker of disease progression (84).

In psoriasis, IL-1ra and IL-1RII were both significantly overexpressed in the suprabasal and basal compartment, respectively, and inversely correlated with the expressions of IL-1α (66). Increased levels of soluble IL-1RII have been found in the synovial fluid (39) and plasma of individuals with RA (78), and these negatively correlated with severity of disease, suggesting IL-1RII acts as natural antagonist of IL-1-driven joint destruction. In contrast, plasma levels of IL-1Ra correlated positively with disease progression, possibly reflecting disease exacerbation (78). These data are in line with experimental *in vitro* and *in vivo* data showing that overexpression of IL-1RII in chondrocytes protected them from IL-1 stimulation (85), or that the transfer of cells overexpressing and releasing IL-1RII resulted in the inhibition of collagen-induced arthritis (52). These results, as well as the binding properties of IL-1RII (high affinity for IL-1, low affinity for IL-1ra), supported the development of IL-1RII as therapeutic molecule in rheumatoid arthritis (see below). The expression of both receptors for IL-1 was demonstrated by immunostaining and laser confocal microscopy in sarcolemma from human muscle tissue samples, at higher levels in patients with polymyositis and dermatomyositis as compared with healthy individuals, together with increased expression of IL-1α, IL-1β, and IL-1Ra (86).

IL-1RII is upregulated in some tumors, including pancreatic ductal adenocarcinoma (87), prostatic cancer and benign prostatic hyperplasia (88), and ovarian cancer, where it provides a powerful distinction between primary and recurrent tumors (89).

In contrast to these conditions associated to upregulation of IL-1RII, in other contexts, defective expression of IL-1RII has been associated to the pathogenesis of the disease. For instance, gene-array analysis of osteoarthritic lesions indicated a lack of expression of IL-1RII and IL-1ra (85), suggesting that defective expression of negative regulators of the IL-1 system contributes to pathogenesis. Similarly, endometriosis and endometrioid ovarian cancer are associated with lower levels of serum and local IL-1RII and with IL-1RII polymorphisms (90– 92). In the context of atherosclerosis, it has been proposed that, since macrophages from hyperlipidemic patients have decreased IL-1RII mRNA and protein expression, IL-1-dependent inflammation could be relatively unchecked during atheroma formation (77). Genome-wide association studies identified several candidate genes potentially involved in inflammatory bowel disease (IBD) pathogenesis, including IL-1RII (91).

Autoimmune inner ear disease is characterized by recurring episodes of sudden or progressive sensorineural hearing loss. Defective responsiveness to corticosteroid in this disease has been correlated to the low induction of IL-1RII in peripheral blood mononuclear cells (93).

Secretion of embryonic IL-1β is one of the first responses of the blastocyst to the receptive endometrium. IL-1β is involved in inducing molecular changes that are essential for attachment of the blastocyst, such as immunomodulation, angiogenesis, and endometrial tissue remodeling. In this context, it has been proposed that these IL-1 activities are regulated by chorionic gonadotropin, which acts directly on endometrial epithelial cells to down-regulate the synthesis and release of IL-1RII (94).

The IL-1 decoy receptor IL-1RII was originally tested as a therapeutic by Amgen in arthritis, based on the promising results in this context (45), but no clinical development of this agent has been reported. Recently, the soluble IL-1RI (Rilonacept) was introduced as therapeutic and approved by the FDA for selected autoinflammatory diseases, in particular cryopyrin-associated periodic syndromes (familial cold autoinflammatory syndrome and Muckle–Wells Syndrome) (95). The drug consists in a fusion protein containing the extracellular domains of IL-1R1 and IL-1RAcP coupled to the Fc region of human IgG1. Rilonacept acts similarly to soluble IL-1RII, as a decoy, by binding IL-1β and IL-1α with higher affinity than IL-1Ra (96).

## The Negative Regulator TIR8/SIGIRR

### Gene and protein

**Figure 2:**
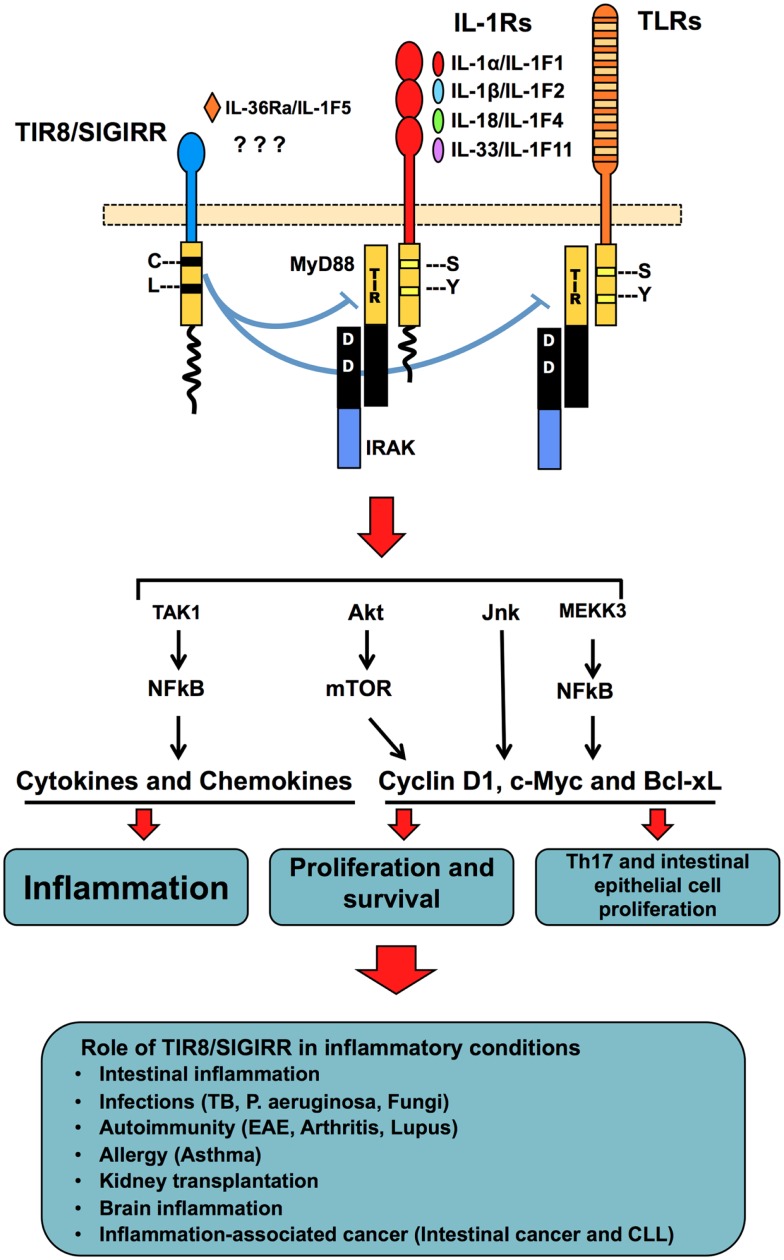
**TIR8/SIGIRR, a negative regulatory receptor of ILR and TLR signaling**. TIR8/SIGIRR is composed by a single extracellular Ig domain, a transmembrane domain, an intracellular conserved TIR domain and a long intracellular tail. Two replaced amino acid in the TIR domain (Cys 222, Leu 305) are potentially involved in non-conventional activation. TIR8/SIGIRR is an orphan receptor, but IL-36Ra has been proposed as a TIR8/SIGIRR ligand in glial cells. TIR8/SIGIRR inhibits ILR and TLR signaling by competing with MyD88 and IRAK recruitment at the TIR domain thus dampening the signaling pathway leading to NF-kB activation. In T cells and epithelial cells, TIR8/SIGIRR inhibits IL-1-dependent activation of the Akt- mTOR pathway and of JNK, thus controlling cell proliferation and survival. *In vivo* studies in gene-targeted mice demonstrate that Tir8/Sigirr acts as a non-redundant negative regulator in different inflammatory conditions dependent on ILRs or TLRs.

TIR8/SIGIRR gene is localized on human chromosome 11 and on murine chromosome 7 (97). The 410 amino acid-long protein is constituted by a single Ig extracellular domain, a transmembrane domain, an intracellular conserved TIR domain, and a 95 amino acid-long tail at the C-terminal, reminiscent of the intracellular tails of few ILR/TLR family members, in particular IL-1AcPb and TIGIRR (see below) (Figure 2). Both in human and mouse, TIR8/SIGIRR has several N- and O-glycosylation sites in the extracellular domain (97, 98). The sequence and pattern of expression of *TIR8/SIGIRR* is conserved among vertebrates, from chicken to humans (99). In particular, human and mouse protein sequences share 82% homology. TIR8/SIGIRR is expressed in several tissues, particularly in kidney, digestive tract, liver, lung, and lymphoid organs (97, 100).

*TIR8/SIGIRR* proximal promoter has a binding site for SP1, which enhances its transcription in basal conditions (101). LPS stimulation reduces SP1 binding to *TIR8/SIGIRR* promoter, possibly explaining the *TIR8/SIGIRR* down-regulation in inflammatory conditions (LPS administration, ulcerative colitis, lung and urinary infections,infestations) (100–106). Recent studies demonstrated lower expression of *TIR8/SIGIRR* in fetal human enterocytes providing a reasonable explanation to the excessive inflammatory response in the immature intestine (107).

In contrast with these studies, *TIR8/SIGIRR* up-regulation was shown in human monocytes during sepsis and sterile systemic inflammation (108). Th2-lymphocytes expressed higher levels of *TIR8/SIGIRR* compared to Th1 polarized or non-differentiated lymphocytes (109). *Pseudomonas aeruginosa* infected mice showed up-regulation of *Tir8/Sigirr* in the cornea, macrophages, and Langerhans cells through the activity of vasoactive intestinal peptide (110). *Lactobacillus jensenii*, a probiotic microorganism, induced up-regulation of *TIR8/SIGIRR* in porcine Payer’s patch antigen presenting cells through activation of TLR2 (111). Similarly, LPS-induced *Tir8/Sigirr* in murine Payer’s patch DCs, but not in spleen DCs (112). These data suggest that Payer’s patch DCs use Tir8/Sigirr to tune TLRs signaling.

### Mechanisms of negative regulation

The function of TIR8/SIGIRR consists in the specific inhibition of NF-kB and JNK activation following stimulation of ILR or TLR family members (102, 113). TIR8 can modulate the signal transduction activated by IL-1RI, IL-18R, T1/ST2, TLR1/2, TLR3, TLR4, TLR7, and TLR9 (98, 102, 109, 113–115) (Figure 2).

The extracellular Ig-like domain of TIR8/SIGIRR has been show to interfere with the dimerization of IL-1RI and IL-1RAcP. The cytoplasmic TIR domain binds TIR-containing adaptor molecules, which are no more available for signaling, whereas the cytoplasmic tail is not involved in the inhibitory activity (102, 114). A computational approach suggests a three-dimensional model for the interaction among the TIR domains of TLR4, TLR7, MyD88, and TIR8/SIGIRR. In this model, TIR8/SIGIRR binds TLR4 and TLR7 through its BB-loop region preventing their dimerization and MyD88 recruitment (116).

TIR8/SIGIRR can also regulate mTOR kinase activity in Th17 lymphocytes (117) and in intestinal epithelial cells (118) (Figure 2). These results are in agreement with the role of TIR8/SIGIRR in autoimmune diseases and in tumor suppression (see below).

### Role of TIR8/SIGIRR *in vivo*

#### Infection-associated inflammation

*Tir8/Sigirr*-deficient mice are more susceptible than wild type mice in several infections, such as tuberculosis, candidiasis, aspergillosis, *P. aeruginosa* infection, in terms of mortality and tissue damage due to an exaggerated inflammatory response (103, 106, 119, 120) (Figure 2). Results obtained with IL-1-blocking antibodies and IL-1RI-deficient mice indicated that in some of these infectious conditions (tuberculosis and *P. aeruginosa* lung infection), TIR8/SIGIRR played a major role in dampening inflammation induced by IL-1R activation.

Similarly, in a colitis mouse model, *Tir8/Sigirr*-deficient mice developed a more severe gut inflammation compared to wild type mice (113, 121). Commensal microflora activates enterocyte TLRs and consequently induces survival of epithelial cells and maintains gut homeostasis (122, 123). Lack of Tir8/Sigirr in colon epithelial cells was shown to be associated to constitutive NF-kB and JNK activation and up-regulated expression of Cyclin D1 and Bcl-xL in homeostatic conditions, which returned to the control level after depletion of commensal bacteria (121) (Figure 2).

Excessive systemic inflammation was observed in *Tir8/Sigirr*-deficient mice upon LPS challenge, and reduced inflammation and mortality were described in Tir8/Sigirr overexpressing mice in LPS-dependent acute lung injury model (102, 124). However, these phenotypes possibly depend on the genetic background since excessive systemic or local inflammatory reactions to LPS were not confirmed in other studies (102, 113).

In contrast with these data, in a urinary tract infection model, Tir8/Sigirr inhibited an effective host response against uropathogenic *E. coli*, as indicated by lower renal bacterial load and dysfunction in TIR8-deficient mice, associated to increased circulating and intrarenal neutrophils at the early phase of infection (125).

#### Sterile inflammatory conditions

Recent data suggest that TIR8/SIGIRR plays a direct role in inhibiting different IL-1-dependent signaling pathways, including IL-1R-mTOR, in Th17 lymphocytes, thus tuning initial Th17 differentiation and preventing Th17 cell-mediated pathogenic effects (117) (Figure 2). This effect was particularly evident in the control of CNS autoimmune inflammation in a model of experimental autoimmune encephalomyelitis (117). Tir8/Sigirr deficiency was also associated to increased susceptibility to develop autoimmunity in a model of systemic lupus erythematosus (B6lpr/lpr), as well as in a model of lupus nephritis induced by hydrocarbon oil (pristane) (115, 126). In the lpr/lpr model, Tir8/Sigirr deficiency was responsible for massive lymphoproliferation, peribronchial inflammation, and mesangio-proliferative glomerulonephritis, due to B and dendritic cell hyper-activation in TLR7- and TLR9-dependent response to autoantigens and nucleosomes (115). Tir8/Sigirr-deficient mice were also more susceptible than wild type mice to both zymosan-induced and collagen antibody-induced arthritis models, because of excessive inflammation at least in part dependent on IL-1 (127) (Figure 2).

In agreement with the results obtained in autoimmunity mouse models, *TIR8/SIGIRR* was down modulated together with other anti-inflammatory genes in psoriatic patients (128).

Studies on allergic inflammatory responses showed that Tir8/Sigirr plays an important role also in controlling the axis IL-33 – ST2 which is involved in Th2 cell polarization and Th2 cytokine production (109) (Figure 2).

DAMPs generated during renal ischemia/reperfusion are responsible of the activation of intrarenal DCs, macrophages, and neutrophils via TLRs and IL-1R, which are potentially involved in post ischemic renal failure. In models of renal ischemia/reperfusion or kidney transplantation, Tir8/Sigirr-deficient mice showed increased renal injury or severe graft rejection, respectively, associated to excessive cytokine and chemokine production and consequently, leukocyte recruitment and amplified adaptive immune response against donor antigens (129, 130) (Figure 2).

Finally, in agreement with the expression in neurons, microglia, and astrocytes (131), TIR8/SIGIRR was shown to be a modulator of microglia activation by LPS, and of neuroinflammation (132). Furthermore, Tir8/Sigirr-deficient mice showed impaired cognitive and synaptic functions associated to up-regulated IL-1R1 and TLR4 signaling in hippocampal tissue in response to IL-1α and high mobility group box 1 (133). Studies on the anti-inflammatory activity of IL-36Ra in the brain demonstrated at least a partial involvement of TIR8/SIGIRR in down modulating glial cell inflammatory responses through the production of IL-4 (18).

#### Cancer-related inflammation

Chronic inflammation is associated with promotion of malignancy and tumor progression and several studies in animals have shown the protumoral role of IL-1 in this context (134, 135). Along the same line, in different murine models, TIR8/SIGIRR has been demonstrated to play a key protective role in the pathogenesis of cancer-related inflammation. In a model of colitis-associated cancer (CAC), a colorectal disease that arises in patients suffering from chronic IBD, Tir8/Sigirr-deficient mice were highly susceptible to both inflammation and carcinogenesis in terms of number, size, and severity of lesions (121, 136) (Figure 2). The mechanism proposed suggests that TIR8/SIGIRR plays a protective role probably by modulating the signaling activated by commensal bacteria through TLRs in the epithelial cells and consequently, downstream events, including production of inflammatory mediators and factors involved in cell survival and proliferation, leukocyte recruitment, and angiogenesis (137). Moreover, Tir8/Sigirr deficiency in the Apc^min/+^ mouse model was associated to increased intestinal lesion development due to higher Akt-mTOR activity, a crucial tumorigenic pathway (118, 138). The data suggest that Tir8/Sigirr exerts a tumor suppressor activity by controlling IL-1- and TLR-induced mTOR-mediated cell cycle progression and consequent genetic instability (118).

In Chronic Lymphocytic Leukemia (CLL), human malignant B cells express lower levels of *TIR8/SIGIRR* mRNA than normal B cells (139, 140). Similar results were found in the mouse where CD19+ cells express lower levels of Tir8 messenger compared to CD19+ cells isolated from a transgenic mouse model of CLL (TCL1 mice) (141). In CLL, both genetic (e.g., MyD88 mutations) and micro environmental factors concur to the development, expansion, and progression of the disease (139, 142). In a murine CLL model, the absence of Tir8/Sigirr led to a more severe and earlier appearance of monoclonal B-cell expansions and to shortened life span. The disease mimicked the aggressive variant of human CLL, characterized by the appearance of prolymphocytes (141) (Figure 2), suggesting that TIR8/SIGIRR acts as an inhibitor of CLL progression through a still unclear molecular mechanism.

## Other ILR with Negative Regulatory Properties

### IL-18 binding protein

IL-18 binding protein (IL-18BP) is a secreted high affinity IL-18 binding molecule, which acts as a potent inhibitor of IL-18 and a modulator of Th1 response. It is constituted by only one Ig-like domain and it is structurally and functionally similar to IL-1RII (30). Indeed, phylogenetic analysis suggests that IL-18BP and IL-1RII had a common ancestral gene and diverged at the level of fish (31). Recombinant IL-1F7 also binds to the IL-18BP, further increasing the ability of IL-18BP to neutralize IL-18 activity (143).

Proteins homolog to IL-18BP have been found in poxviruses (*Ectromelia*), which are responsible of neutralization of human IL-18 during the viral infection and of dampening the inflammatory response associated to the infection (144).

Further information about this molecule is available in the review by Dinarello et al. in this issue.

### IL-1RAcPb

The IL-1RAcP is the receptor subunit of the IL-1RI complex, and it is also used by IL-36α/IL-1F6, IL-36β/IL-1F8 and IL-36γ/IL-1F9, and IL-33 receptors. It has been shown that an alternative form of AcP, called AcPb, can be generated by alternative splicing, in which the prototypical AcP C-terminal exon 12 is skipped and an alternative exon 12b is used (145, 146). Smith et al. (146) characterized this molecule and showed its regulatory properties in the brain. The C-terminus encoded by these two alternative exons has 35% amino acid identity, which includes conserved motifs of the TIR domain. Moreover, the exon 12b encodes a sequence of approximately 140 additional amino acids in the C-terminal of the TIR domain that has no homology to other protein sequences and is of unknown function. The general structure of AcPb is similar to that of AcP and suggests that the AcPb cytoplasmic domain is similar to AcP TIR domain. However, there is one area of substantial difference because of changed configuration in the DD loop and aD helix regions of the AcPb TIR domain, which remembers modification of TIR8/SIGIRR TIR domain, and altered charge distribution pattern on its surface. It has been proposed that these modifications could affect interaction with adapter and signaling molecules. Indeed, AcPb is capable of forming a ligand-dependent complex with IL-1R, but it does not lead to the recruitment of the adaptor molecules MyD88 and IRAK4 after stimulation with IL-1, and is unable to mediate specific IL-1 responses. In both human and mouse, the expression of the AcPb is restricted to the CNS (whole brain, fetal brain, cerebellum, and spinal cord). AcP and AcPb are coexpressed in the same cells, but AcPb is the more abundant isoform. It has been proposed that AcPb could also be recruited to other AcP-utilizing receptors, such as ST2 and IL-1Rrp2/IL-36R, which are expressed in the CNS, once they have bound their ligands (IL-33 and IL-36α,β,γ, respectively). In a model of LPS challenge in the CNS, AcPb-deficiency was associated to neuronal loss suggesting that AcPb may dampen the neurotoxic effects of IL-1 by modulating the intracellular signaling and gene expression response to LPS-induced IL-1, or possibly to other cytokines acting through AcP (146). The inhibitory effect of AcPb could depend on the failure to recruit MyD88 and IRAK4, on the competition with AcP in a IL-1 receptor complex containing multiple IL-1R and AcP molecules, or on unknown functions mediated by the C-terminal tail.

### TIGIRR-1 and IL-1RAPL

TIGIRR-1 and IL-1RAPL (also named TIGIRR-2) are localized on the X chromosome and share between 22 and 48% overall identity to other ILR family members. IL-1RAPL and TIGIRR-1 exons are spread out over a very large segment of genomic DNA (more than 1500 kb for IL-1RAPL and 380 kb for TIGIRR-1). Both TIGIRR-1 and IL-1RAPL contain a signal peptide, three predicted extracellular Ig domains, a single transmembrane domain, and a highly conserved cytoplasmic region containing a C-terminal cytoplasmic extension reminiscent of the *Drosophila* Toll family, TIR8/SIGIRR, and AcPb cytoplasmic domains.

A negative regulatory role has not yet been reported for these two receptors. However, in *in vitro* studies performed with chimeric molecules, the cytoplasmic domains of TIGIRR-1 and IL-1RAPL fused to the extracellular and transmembrane domains of IL-1RI or AcP could not induce NF-κB, similarly to TIR8/SIGIRR, and in contrast with the cytoplasmic domains of other members of the ILR family (10). Other functional studies showed that IL-1RAPL can activate JNK but not the ERK or the p38 MAP kinases, whereas TIGIRR-1 cannot activate JNK. Deletion mutagenesis studies showed that the activation of JNK by IL-1RAPL does not depend on the integrity of its TIR domain, suggesting a distinct mechanism of signaling through this receptor (147).

TIGIRR-1 is highly conserved in human and mouse (94.5% identical at the amino acid level) and it is expressed in skin, liver, placenta, and fetal brain. IL-1RAPL, whose crystal structure has been determined (147), is expressed in heart, brain, ovary, skin, and to a lesser extent in tonsil, fetal liver, prostate, testis, small intestine, placenta, and colon. IL-1RAPL was identified as the gene responsible for hereditary non-syndromic mental retardation and autism linked to chromosome region Xp22.1–21.3 (148, 149). It is expressed in brain structures involved in the hippocampal memory system, and it has a role in brain development and function (150). No information are available about a potential role of IL-1RAPL in inflammation and defense, however, its C-terminal extension is reported to interact with neuronal calcium sensor-1 and regulate neurite outgrowth (150–152).

### DIGIRR

Recently, a new member called DIGIRR was added to the ILR family (153). DIGIRR was discovered in teleost fish and showed high homology with TIR8/SIGIRR. DIGIRR is characterized by an extracellular portion comprising two Ig-like domains, a transmembrane domain and TIR domain carrying two amino acid substitutions (Arg419-Tyr420), which are responsible for the loss of signaling. The DIGIRR mRNA was found expressed in several tissues and in leukocytes and was upregulated by LPS, oppositely to TIR8/SIGIRR, suggesting a different mechanism of response to inflammatory stimuli between the two molecules. At the subcellular level, DIGIRR showed a peculiar distribution within the Golgi apparatus.

Different lines of evidence suggest that DIGIRR acts as negative regulator of LPS- and IL-1β-induced inflammation. Indeed, siRNA knock down of DIGIRR lead to increased production of IL-β-induced pro-inflammatory cytokines in liver, kidney, and leukocytes. Moreover, *in vitro* administration of DIGIRR to zebrafish embryos significantly inhibited LPS- and IL-1β-induced activation of NF-kB.

The discovery of DIGIRR could help to understand the evolution of ILR family members. Indeed, the authors suggest the hypothesis that DIGIRR and TIR8/SIGIRR derive from a common ancestral molecule that lost respectively one or two Ig-like extracellular domains, and Ser or Arg-Tyr- amino acids in the TIR domain. DIGIRR might represent an evolutionary intermediate molecule between IL-1R and TIR8/SIGIRR, demonstrating a shift from a potent receptor to a negative regulator.

## Concluding Remarks

Studies conducted in the early 1990s suggesting that the non-signaling IL-1RII acts as a molecular trap for the agonist and the AcP, led to the formulation of the decoy paradigm, which has then been extended to other cytokine families and chemokines. Decoy receptors are now recognized as a general strategy to tune the actions of primary inflammatory cytokines and chemokines.

The list of ILR/TLR family receptors acting as negative regulators now includes TIR8/SIGIRR, which acts by modulating ILRs- or TLRs-dependent signaling. In addition to these molecules, soluble forms of signaling receptors or AcP act as decoys or negative regulators by trapping the ligands. For instance, T1/ST2 exists also as a soluble isoform obtained by differential mRNA processing, which acts as an antagonistic decoy receptor for IL-33 (154), and has been proposed in the therapy of arthritis (155). Similarly, soluble IL-1AcP, generated by alternative splicing, forms a complex with IL-1β and IL-1RII playing a protective role in arthritis (156) and has been pharmacologically exploited.

For several of these molecules further studies have to be performed to unequivocally define their role in disease and their potential as therapeutic targets. For instance, unfortunately there are no genetic evidence on the consequence of IL-1RII-gene deficiency or data supporting the relevance of TIR8/SIGIRR in human disease. In addition, the clinical development of IL-RII pharmacological targeting has not been reported. Finally, pharmacological approaches targeting TIR8/SIGIRR functions have not been developed yet and they will be necessary to assess whether TIR8/SIGIRR might be a therapeutic target in inflammatory conditions.

However, the existence of IL-1RII, together with IL-1Ra, TIR8/SIGIRR, brain AcPb, and other soluble receptors acting as molecular traps emphasizes the need for tight control of the IL-1 system, which mediates potentially devastating local and systemic inflammatory reactions.

## Conflict of Interest Statement

The authors declare that the research was conducted in the absence of any commercial or financial relationships that could be construed as a potential conflict of interest.
